# Long‐Term Active Rather than Passive Restoration Promotes Soil Organic Carbon Accumulation by Alleviating Microbial Nitrogen Limitation in an Extremely Degraded Alpine Grassland

**DOI:** 10.1002/advs.202510549

**Published:** 2025-11-29

**Authors:** Jinchao Gong, Feida Sun, Jingjing Wu, Shijie Zhou, Yue Xiu, Linlin Li, Tahmina Kausar, Zhouwen Ma, Jiqiong Zhou, Lin Liu, Yongxing Cui, Jordi Sardans, Josep Peñuelas, Ahmed Elrys, Ji Chen, Yanfu Bai

**Affiliations:** ^1^ College of Grassland Science & Technology Sichuan Agricultural University Chengdu Sichuan 611130 P. R. China; ^2^ Institute of Biology Freie Universität Berlin 14195 Berlin Germany; ^3^ CSIC Global Ecology Unit CREAF‐ CSIC‐UAB Bellaterra 08193 Catalonia Spain; ^4^ CREAF Cerdanyola del Vallès Barcelona 08193 Catalonia Spain; ^5^ College of Agriculture University of Al Dhaid Al Dhaid Sharja United Arab Emirates; ^6^ College of Tropical Agriculture and Forestry Hainan University Haikou Hainan 570228 China; ^7^ State Key Laboratory of Loess and Quaternary Geology Institute of Earth Environment Chinese Academy of Sciences Xi'an Shaanxi 710061 P. R. China

**Keywords:** active restoration, alpine grassland, carbon use efficiency, microbial nitrogen limitation, passive restoration, soil enzyme activity

## Abstract

Grassland degradation disrupts microbial nutrient cycling, yet the role of nitrogen (N) limitation in regulating soil organic carbon (SOC) dynamics during restoration remains poorly understood. Here, 10 years of active (sowing of seeds of native plants) and passive restoration (sand barrier protection) in degraded grasslands on the Qinghai–Tibetan Plateau are compared. Restoration impacts are assessed by integrating microbial metabolic traits such as stoichiometry‐based nutrient limitation and C use efficiency (CUE_ST_) with SOC fractionation, which considers both POC and MAOC). Active restoration reduces microbial N limitation by 44–71%, driving a 291–467% increase in SOC stocks, from 0.81 to 3.15 kg m^−2^ in topsoil and 0.54 to 3.08 kg m^−2^ in subsoil. It also reduces CUE_ST_ by 54% in topsoil and 34% in subsoil, boosting POC by 483–557% and MAOC by 621–1,071%. MAOC dominates SOC accumulation, exceeding POC by 2.3–7.2 times. The CUE_ST_ reduction aids POC transformation into MAOC, stabilizing SOC storage. In contrast, passive restoration slightly reduces N limitation by 36–39% and CUE_ST_ by 10–23%, but failed to enhance C fractions or SOC stocks due to persistent nutrient constraints. The findings demonstrate that alleviating microbial N limitation by active restoration is critical for stabilizing SOC through MAOC accumulation.

## Graphical abstract

Active restoration increases soil organic carbon stocks by reducing microbial nitrogen limitation. Nitrogen availability promotes particulate to mineral‐associated organic carbon conversion by reducing microbial carbon use efficiency. Passive restoration has no effect on soil organic carbon stocks.

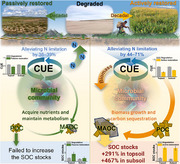

## 1 Introduction

Grasslands play a critical role as terrestrial carbon (C) sinks, containing roughly 20% of the world's soil organic C (SOC).^[^ ^1^, ^2^ ^]^ However, approximately half of all global grasslands are degraded, and on the Qinghai‐Tibetan Plateau (QTP), the degradation exceeds 90%.^[^ ^3^, ^4^ ^]^ Degradation typically reduces vegetation cover, productivity, and SOC.^[^ ^5^ ^]^ Strategies for preventing grassland degradation and restoring soil C stocks have attracted extensive scientific attention.

Grassland restoration can be achieved through active or passive approaches.^[^ ^6^ ^]^ Active restoration employs direct interventions, including planting, seeding, soil amendments, and supplementary management techniques, to accelerate ecosystem recovery. For example, seeding in degraded areas overcomes seed dispersal limitations and promotes more effective restoration.^[^ ^7^ ^]^ Passive grassland restoration, sometimes referred to as natural regeneration, is characterized by minimal human involvement. This approach focuses on removing the primary drivers of ongoing degradation, enabling the ecosystem to recover naturally through existing seed banks and ecological processes.^[^ ^8^ ^]^ Studies have demonstrated that the relative effectiveness of active vs passive restoration can depend on conditions. Both active and passive restoration approaches have enhanced soil C storage;^[^ ^6^, ^9^ ^]^ active restoration tends to enhance SOC to a greater extent in severely degraded, C‐poor soils and topsoil, whereas passive restoration can be more effective in C‐rich soils and subsoil horizons.^[^ ^6^ ^]^ Additionally, active restoration often restores degraded grasslands faster than passive restoration. For instance, multispecies plantings restored SOC stocks within 13‐17 years,^[^ ^10^ ^]^ whereas passive succession may require many decades to return to pre‐degradation levels.^[^ ^11^, ^12^, ^13^ ^]^ These observations suggest that the outcome of restoration strategies depends on specific environmental factors which influence how quickly C stocks can recover. However, the processes by which active and passive restoration strategies facilitate C sequestration have yet to be fully clarified.

The nutrient status of soil, especially nitrogen (N), strongly regulates microbial C use efficiency (CUE), which influences how C is partitioned between the labile and stable soil pools. CUE is the fraction of C used by microbes for biomass growth, rather than being respired.^[^ ^14^ ^]^ Under N limitation, microbes must divert more C to produce N‐acquiring enzymes and other nutrients, which lowers CUE and causes more C to be lost as CO_2_.^[^ ^15^ ^]^ Conversely, when N is abundant, microbes can invest a greater share of substrate C into growth, raising CUE and reducing respiratory C losses.^[^ ^16^ ^]^ SOC occurs in two contrasting forms: particulate organic C (POC) and mineral‐associated organic C (MAOC). POC consists of relatively labile debris and biomass, while MAOC is the much more persistent fraction that is physically or chemically bound to soil minerals.^[^ ^17^ ^]^ Importantly, high microbial CUE tends to drive more of the decomposed C into microbial biomass that can become stabilized as MAOC, whereas low CUE leaves more C respired or retained as transient POC. For example, Guo et al. (2025) reported that adding N‐fixing legumes to grassland lowered the soil's C:N ratio and raised microbial CUE, which led to an increase of 5.7% in total SOC and 7.4% in MAOC when compared to the grassland areas that lacked legumes.^[^ ^18^ ^]^ Overall, microbial CUE provides a key mechanism connecting soil nutrients to SOC stabilization.

Grassland restoration practices alter the way nutrients affect CUE and C partitioning. Active restoration increases soil N rapidly and relieves the N stress that microbes experience.^[^ ^19^ ^]^ For instance, adding N‐fixing legumes or seeding degraded grassland improves soil fertility and N availability, which boosts microbial CUE and accelerates C turnover. Roa‐Fuentes et al. (2013) demonstrated that planting legumes enhanced N cycling and microbial biomass N (MBN), whereas grazing exclusion mainly enriched labile C and N pools, such as litter and microbial biomass C (MBC).^[^ ^20^ ^]^ Similarly, Lu et al. (2023) found that seeding in an arid steppe yielded higher soil‐quality indices than simple exclusion, indicating faster recovery of soil C and nutrients.^[^ ^21^ ^]^ In contrast, passive restoration, which enables natural vegetation recovery, raises N and soil fertility more gradually. Initially, in this natural process, litter and microbial biomass build up, leading to higher POC levels. Since decomposition is slow, the soil contains more labile C at first.^[^ ^20^ ^]^ Over time, nutrient cycling and CUE increase under passive recovery, but if N remains limiting for long periods, MAOC formation can lag, potentially leaving SOC more vulnerable to loss. Therefore, the key factors regulating the effectiveness of active versus passive restoration for soil C sequestration remain uncertain. Addressing this gap is essential for predicting the long‐term SOC outcomes of grassland recovery. However, despite the recognized importance of microbial N limitation in SOC dynamics, the mechanisms linking N availability, microbial CUE, and SOC stabilization during restoration remain poorly understood.^[^ ^22^ ^]^ This gap persists because most studies have either focused on short‐term restoration,^[^ ^23^ ^]^ lacked concomitant measurements of microbial CUE and SOC fractions and/or did not compare active versus passive restoration under highly degraded conditions.^[^ ^24^ ^]^ Specifically, few field‐based studies have investigated the link between restoration and carbon (C) redistribution in a mechanistic way. This gap includes comparing how active and passive restoration in severely degraded alpine systems affect microbial N limitation, C use efficiency (CUE), and the transformation of C between particulate organic C (POC) and mineral‐associated organic C (MAOC) across soil depths.

To elucidate the origins of these differences, microbial CUE, C pools, and nutrient limitations were assessed in extremely degraded grasslands subjected to both passive and active restoration on the QTP for at least a decade. We measured soil microbial CUE (using a stoichiometry‐based community‐level estimate, CUE_ST_), SOC fractions (POC and MAOC), and nutrient limitations.^[^ ^25^, ^26^ ^]^ The following questions were addressed: 1) does long‐term active restoration lead to greater SOC accumulation than passive restoration in extremely degraded alpine grasslands, and 2) does active restoration alleviate microbial N limitation and enhance CUE, leading to a greater conversion of POC to MAOC, more effectively than passive restoration? We propose the following hypotheses: i) active restoration results in greater SOC accumulation than passive restoration after at least 10 years of implementation in extremely degraded alpine grasslands; ii‐a) active restoration alleviates microbial N limitation more effectively than passive restoration; and ii‐b) as a consequence of reduced N limitation and enhanced microbial activity, active restoration accelerates the microbial transformation of labile POC into stable MAOC to a greater extent than passive restoration, resulting in greater SOC stability. By linking microbial stoichiometry (CUE) and nutrient limitation to SOC fractions, this study provides new mechanistic insight into how restoration strategies regulate long‐term C sequestration in alpine grasslands.

## 2 Results

### 2.1 Effects of Restoration Type on Soil C Cycling

**Figure 1:**
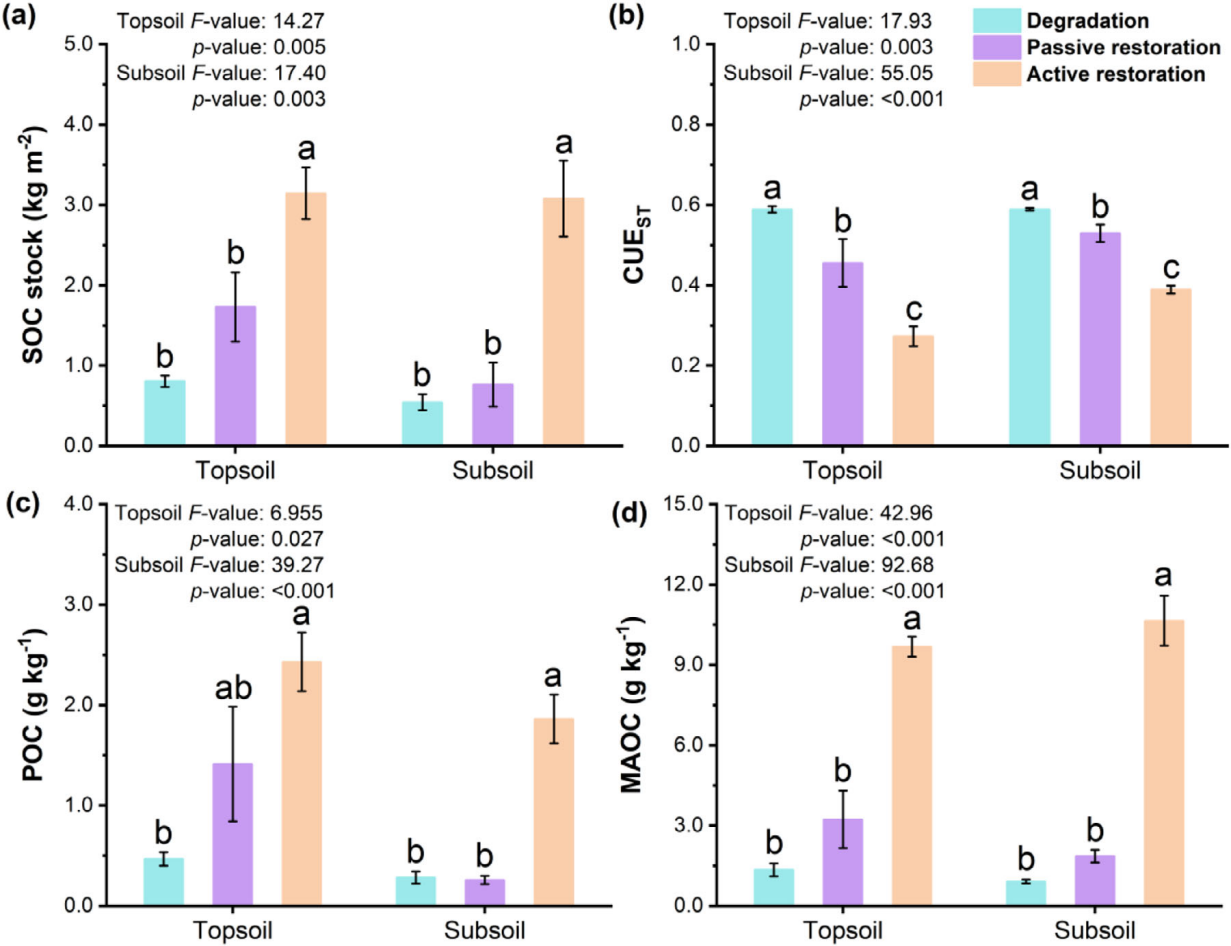
The effects of active and passive restoration on a) soil organic C stocks, b) soil microbial C use efficiency (CUE_ST_), c) particulate organic C (POC), and d) mineral‐associated organic C concentration (MAOC). Data are presented as mean ± SE. Means with different letters differ from each other (*p* < 0.05).

Actively restored grassland had greater (*p* < 0.05) SOC stock than degraded grassland ‐ by 291% in the topsoil and 467% in the subsoil (**Figure** **1**a); however, SOC stocks did not differ (*p* > 0.05) between passively restored and degraded grasslands (Figure 1a). CUE_ST_ declined with both restoration strategies, displaying a moderate decrease with passive restoration of 23% in topsoil and 10% in subsoil, but a significant decrease (*p* < 0.05) with active restoration of 54% in topsoil and 34% subsoil (Figure 1b). These patterns emphasize the distinct impacts of active and passive restoration on SOC dynamics and microbial functionality.

POC and MAOC were greater (*p* < 0.05) in actively restored than degraded grasslands by 483% and 557% in the topsoil and 621% and 1071% in the subsoil, respectively (Figure 1c,d). In contrast, POC and MAOC did not differ (*p* > 0.05) between passively restored and degraded grasslands (Figure 1c,d).

### 2.2 Soil Enzyme Activity and Microbial Nutrient Limitation

Phosphorus‐acquisition enzyme acid/alkaline phosphatase (AP) was greater (*p* < 0.05) in actively restored than degraded grasslands by 2689% in topsoil and 5711% in subsoil; C‐acquisition enzymes β‐glucosidase (βG) and cellobiohydrolase (CBH) were greater by 1770% and 8470% in topsoil, and 8647% and 13950% in subsoil, respectively; and N‐acquisition enzymes leucine aminopeptidase (LAP), β‐N‐acetylglucosaminidase (NAG), and urease (UE) were greater by 208%, 367%, and 977% in the topsoil and subsoil (Figure S1a, Supporting Information). In contrast, extracellular enzyme activity (EEA) did not differ between passively restored and degraded grasslands (Figure S1, Supporting Information). Degraded grassland had greater EEA_N:P_ than the restored grasslands, indicating elevated microbial N demand (Figure S1, Supporting Information).

Topsoil and subsoil EEA_C:N_, were greater by 484% and 2428%, respectively, in actively restored than degraded grasslands, reflecting heightened microbial C demand. In passively restored grassland, EEA_C:N_ in the subsoil was 1373% greater (*p* < 0.05) than in degraded grassland (Figure S1b, Supporting Information). The enzyme vector angles were greater (*p* < 0.05) in passively restored (topsoil: 42.1, subsoil: 36.0) than degraded grassland (topsoil: 30.8, subsoil: 25.9) by 36% and 39% in the topsoil and subsoil, respectively; and were greater (*p* < 0.05) in actively restored (topsoil: 44.4, subsoil: 44.2) than degraded grassland by 44% and 71% in the topsoil and subsoil, respectively, Figure S1a, Supporting Information). This suggests that, compared to degraded grassland, both passive and active restoration had lesser microbial N limitation.

**Figure 2:**
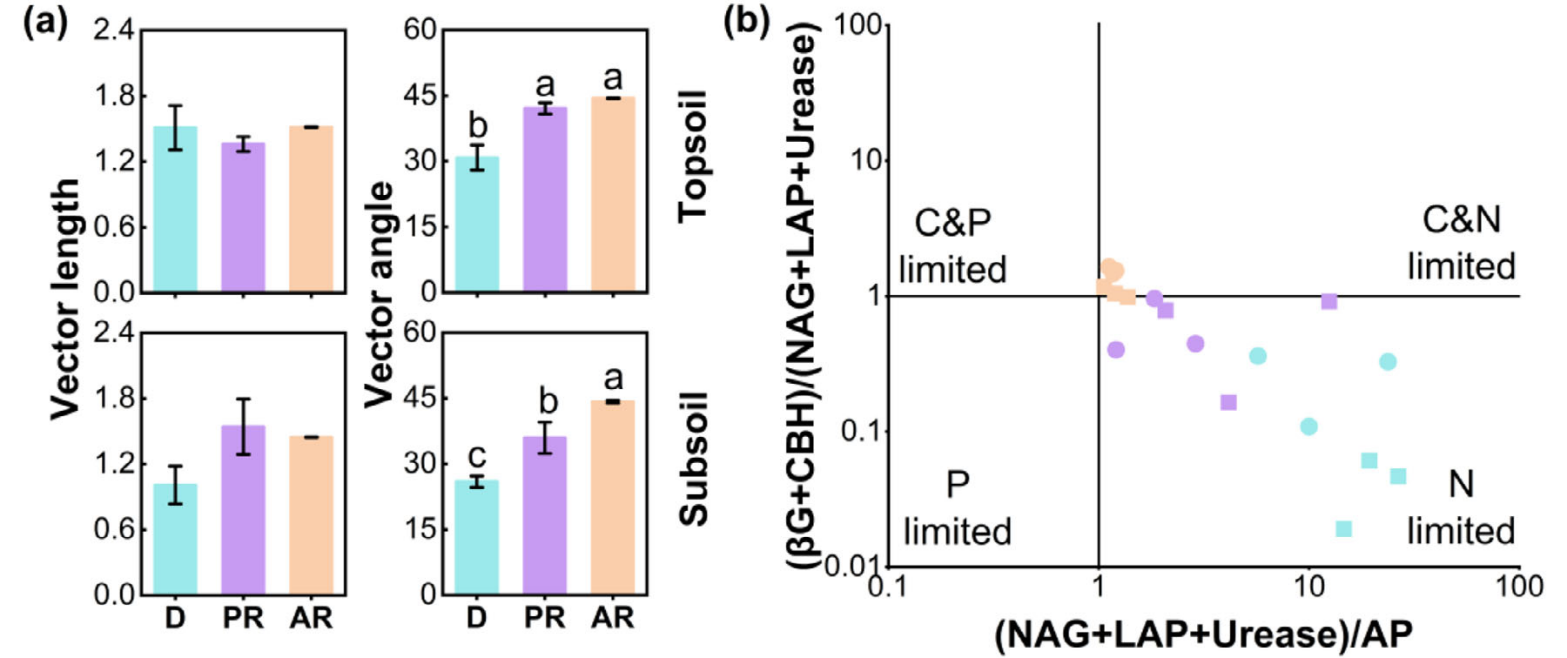
a) The effects of active and passive grassland restoration on soil enzyme vectors (means ± SE). b) Scatter plot depicting microbial resource constraints based on soil enzymatic stoichiometry. Means with different letters differ from each other (*p* < 0.05). D: degraded, PR: passive restoration, AR: active restoration.

Relative to the degraded site, both restoration approaches altered enzyme stoichiometric ratios, which reflect microbial nutritional status (**Figure** **2**b). To assess microbial nutrient limitation in the soil, we used nutritional limitation parameters based on ecological enzyme activity stoichiometry. Vector analysis provides a relative measure of nutrient limitation: longer vector lengths indicate greater C limitation, angles greater than 45° indicate P limitation, and angles less than 45° indicate N limitation.^[^ ^27^ ^]^ In the degraded grassland, N limitation for soil microbes was relatively severe in both topsoil and subsoil, but it was alleviated under both passive and active restoration (Figure 2a,b). Notably, while active restoration reduced the degree of microbial N limitation, it slightly increased C limitation (Figure 2b).

### 2.3 The Drivers of SOC Stocks

**Figure 3:**
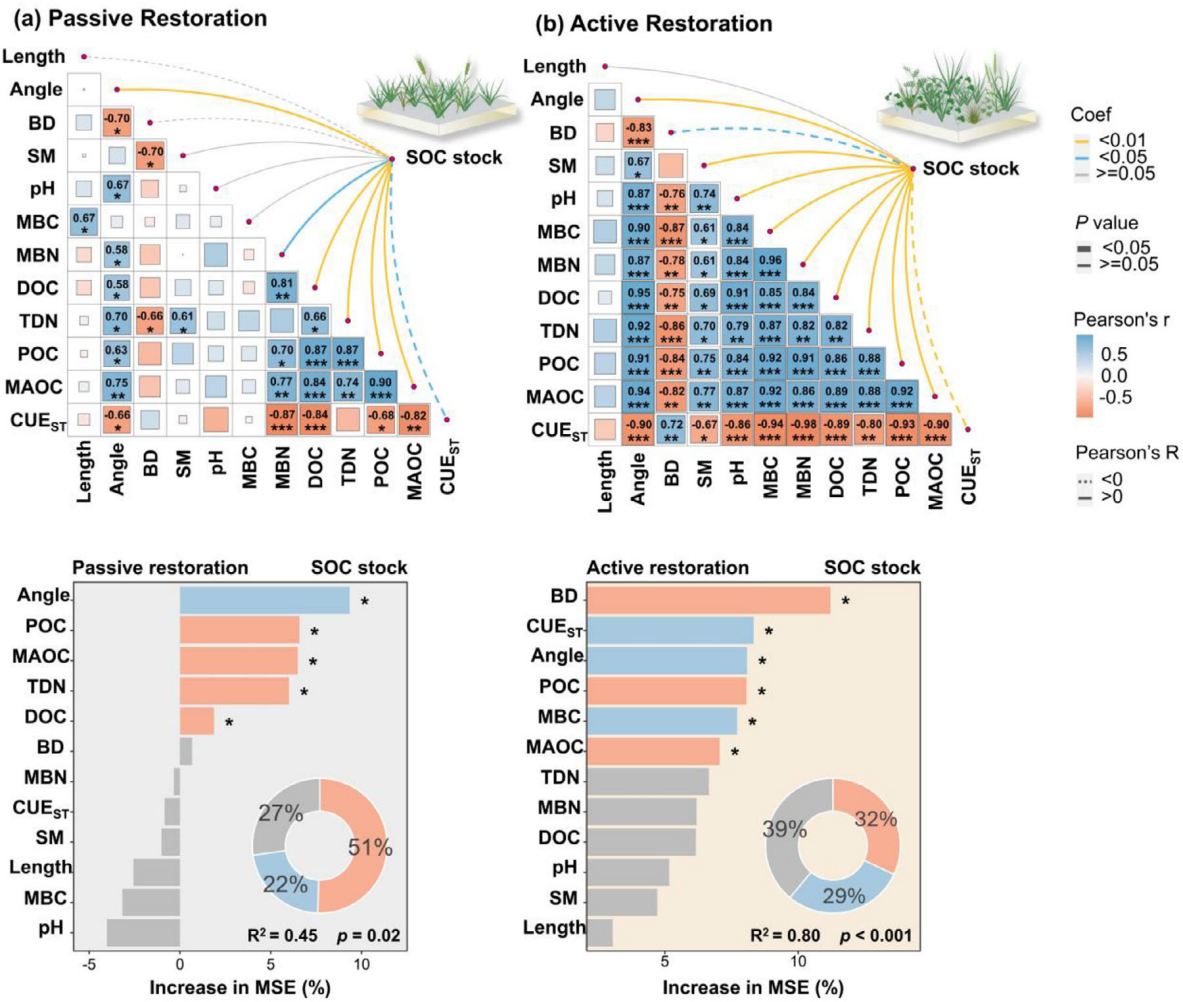
Correlations between soil enzyme vectors, SOC stocks, SOC component pools (POC and MAOC), and soil properties under passive a) and active c) restorations. Random forest modelling estimates of the importance of predictors of soil enzyme vector angle, microbial CUE_ST_, and SOC components on SOC stocks under passive b) and active d) restorations. The insets (the circle) in (c) and (d) represent the relative contributions of soil (purple), microbial (blue), and irrelevant (gray) to the variability of SOC stocks in passive and active restorations. ^*^: *p* < 0.05, ^**^: *p* < 0.01, ^***^: *p* < 0.001. The relative importance of predictors was estimated by the percentage increase in the mean square error (%MSE). Significance levels: ^*^ *p* < 0.05. Network graphs displaying Pearson correlation coefficients between variables. Length: soil enzyme vector length, Angle: soil enzyme vector angle, CUE_ST_: microbial C use efficiency, BD: bulk density, SM: soil moisture, pH: soil pH, MBC: soil microbial biomass C, MBN: soil microbial biomass N, DOC: soil dissolved organic C, TDN: soil total dissolved N, POC: particulate organic C, MAOC: mineral‐associated organic C.

To test linear relationships between microbial and soil variables, we performed Pearson correlation analysis between microbial CUE_ST_, enzyme vector angle and length, POC, MAOC, and SOC stocks. Under both restoration approaches, SOC stocks were correlated positively with enzyme vector angle (*p* < 0.01), POC (*p* < 0.01), and MAOC (*p* < 0.01), but negatively with microbial CUE_ST_ (passive restoration, *p* < 0.05, active restoration, *p* < 0.01, **Figure** **3**a,b). Additionally, soil enzyme vector angle, POC, and MAOC were correlated positively (*p* < 0.05) with both passive and active restoration, while CUE_ST_ was correlated negatively (*p* < 0.05) with all three variables (Figure 3a,b). Soil enzyme vector angle was correlated positively with SOC stocks, total dissolved nitrogen (TDN), microbial biomass carbon (MBC), and microbial biomass nitrogen (MBN), but negatively with soil bulk density (BD) (Figure 3b).

To identify and rank the most important predictors of SOC stocks under each restoration type, we employed a random forest model with 1000 trees, including POC, MAOC, bulk density (BD), CUE_ST_, and enzyme vector metrics. Based on random forest modeling, vector angle and POC were the most important factors influencing SOC stocks, followed by MAOC, TDN, and DOC in passive restoration (Figure 3c), while BD, CUE_ST_, and vector angle were the main factors driving SOC stock, followed by POC, MBC, and MAOC in active restoration (Figure 3d).

**Figure 4:**
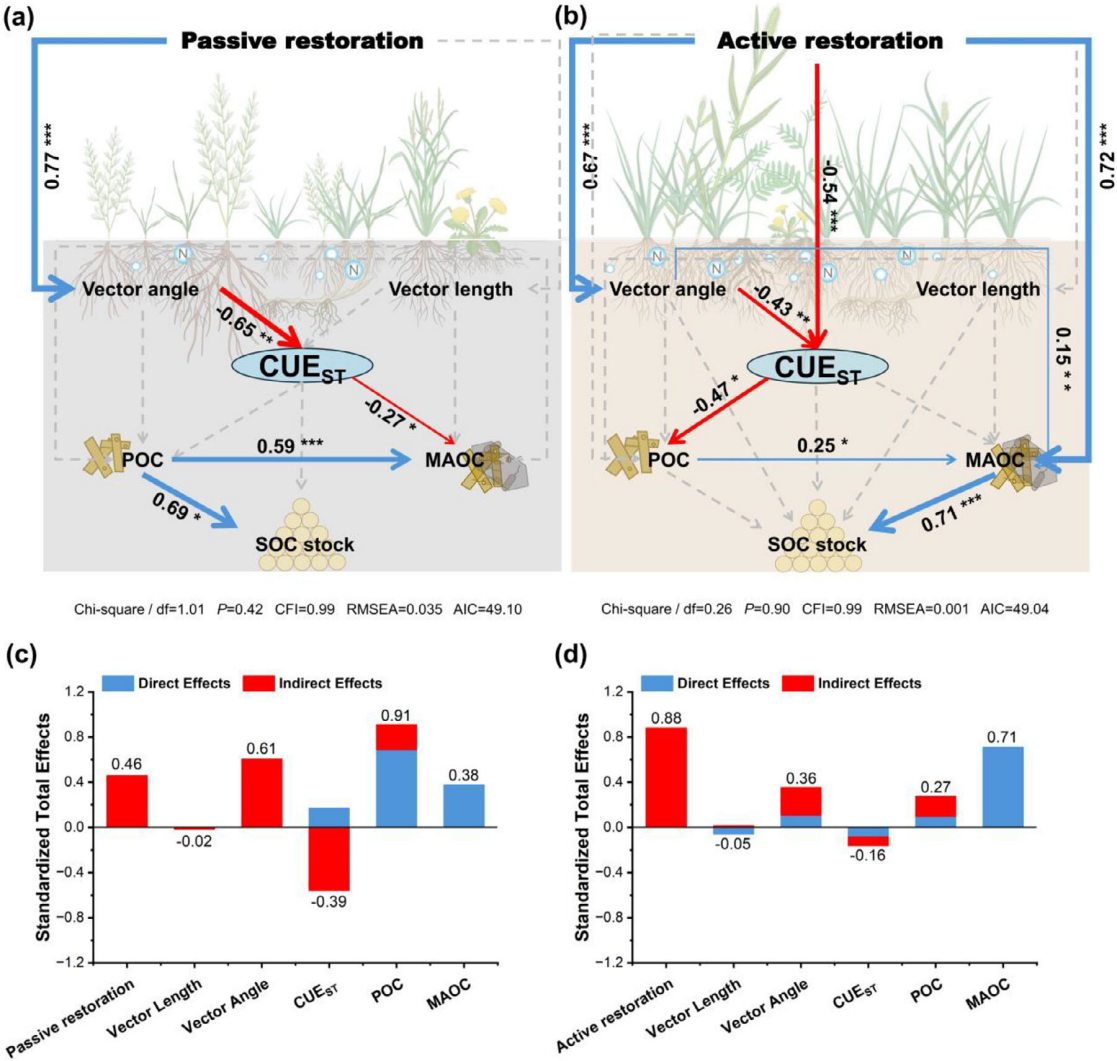
Structural equation model (SEM) examining the standard total effects of soil enzyme vector angle, vector length, CUE_ST_, POC, and MAOC on SOC stocks under passive and active restoration. Solid red and blue arrows represent negative and positive standard path coefficients (*p* < 0.05), respectively, and dotted gray arrows represent non‐significant paths (*p* > 0.05). The thickness of the arrows indicates the strength of the path coefficients. R^2^ denotes the proportion of variance explained. ^*^, ^**^, and ^***^ indicate significance at *p* < 0.05, *p* < 0.01, and *p* < 0.001.

To test hypothesized causal pathways linking microbial N limitation, microbial CUE, SOC fractions, and total SOC, we generated a structural equation model (SEM) and evaluated model fit using chi‐square statistics. The SEM analysis revealed that lower CUE_ST_ was driven by enzyme vector angle, indirectly increasing SOC stocks under active restoration. In active restoration, the reduction in CUE_ST_ facilitated SOC stock increases by promoting POC accumulation and its transformation into MAOC (**Figure** **4**).

## 3 Discussion

Active restoration increases SOC in degraded alpine grasslands to a greater extent than passive restoration, mainly due to a substantial rise in MAOC in subsoil under active restoration. Enhanced CUE_ST_, driven by reduced microbial N limitation, promoted the conversion of POC into MAOC. Although the reduced CUE_ST_ altered the way microbes allocated resources, it was linked to an increase in both microbial biomass and the formation of MAOC. In contrast, passive restoration alleviated N limitation only slightly and failed to increase SOC, POC, or MAOC. These findings indicate that, by addressing microbial nutrient deficits, active restoration stabilized more C in the soil. This study integrates measurements of SOC fractions, microbial enzyme stoichiometry, and CUE, providing new insights into the mechanisms by which active versus passive restoration influences soil C accumulation.

### 3.1 Active, Rather than Passive, Restoration Enhanced SOC Stocks by Alleviating Soil Microbial N Limitation

Active restoration generally led to faster and larger gains in soil C and nutrient status than passive restoration. For example, global studies reported that restoration of degraded sites increased SOC substantially more than the degraded state^[^ ^24^ ^]^ while meta‐analyses concluded that active measures tended to boost SOC more than passive measures.^[^ ^28^ ^]^ In dryland meta‐analyses, active restoration consistently improved soil C, whereas passive soil restoration often produced only weak or even negative gains.^[^ ^25^, ^28^ ^]^ In the present study, active restoration increased SOC stocks, consistent with previous studies demonstrating its effectiveness for C storage.^[^ ^29^ ^]^ This increase was driven by the alleviation of microbial N limitation, as both passive and active restoration reduced N constraints, but active restoration did so more effectively. Active restoration in the present study included planting the leguminous species *Melissitus ruthenica*, which was still present during our survey (Table S1, Supporting Information). The presence of this legume likely contributed to biological N fixation in the soil, providing additional N inputs that helped relieve microbial N limitation and supported greater microbial activity and SOC stabilization.^[^ ^30^ ^]^ This was supported by strong correlations between soil enzyme vector angle (indicative of reduced microbial N limitation) and increases in SOC stocks, TDN, MBC, and MBN, as well as reductions in soil BD. In addition, soil bulk density was reduced with active restoration, likely reflecting greater root activity and soil aggregation, which can improve soil structure and enhance plant productivity, further boosting C inputs.^[^ ^31^ ^]^

In the present study, both restoration treatments reduced microbial N‐limitation (as indicated by enzyme stoichiometry), but the effect was stronger under active than passive restoration. This agrees with other reports that vegetation restoration boosts N‐cycling enzymes and soil N availability.^[^ ^32^ ^]^ For example, grassland restoration can increase N‐acquiring enzyme activities, which enable microbes to overcome N scarcity. Because active restoration typically accelerates plant growth and organic inputs, it tends to build up total soil N (e.g., total dissolved N, microbial N biomass, Table S2, Supporting Information) more rapidly than passive recovery.

Enzyme stoichiometry further reflected shifts in microbial nutrient demands. Active restoration increased extracellular enzyme activities (EEAs) associated with C, N, and P cycling to a greater extent than degraded soils.^[^ ^33^ ^]^ However, this increase in EEAs came at the cost of reduced microbial CUE_ST_, as microbes allocated more resources to enzyme production to acquire C and nutrients, thereby facilitating the transformation of POC into MAOC.^[^ ^25^, ^34^ ^]^ Such a trade‐off between CUE and enzyme investment is consistent with ecological theory and has been observed in other studies,^[^ ^35^ ^]^ where microbes facing nutrient limitation increase resource acquisition at the expense of growth efficiency. Increased soil moisture in the topsoil (Table S2, Supporting Information), a critical limiting factor in degraded grasslands, further amplified these effects by enhancing microbial activity.^[^ ^36^ ^]^ Active restoration increased root biomass and litter cover, which improved soil water retention^[^ ^37^ ^]^ and further amplified enzyme activity. The reduction in CUE under active restoration thus played a crucial role in promoting stable SOC storage through MAOC accumulation. Greater microbial biomass (MBC and MBN) and EEAs under active restoration indicated increased microbial processing of organic matter,^[^ ^30^, ^38^ ^]^ as was evidenced by the dominance of MAOC in SOC accumulation. Microbial enzymes facilitated the breakdown of organic matter and its aggregation with soil minerals, forming stable MAOC.^[^ ^39^ ^]^ These processes could explain the increase in subsoil SOC, as MAOC is inherently more stable than POC.

### 3.2 Mechanisms Linking N Limitation Alleviation and C Stabilization

Active restoration increased SOC stocks primarily through increases in MAOC, which aligns with studies emphasizing mineral‐associated C as a key stable pool.^[^ ^40^ ^]^ A notable finding was the negative correlation between microbial CUE_ST_ and SOC stocks, while isotope‐based studies often reported a positive CUE‐SOC relationship.^[^ ^14^, ^41^, ^42^ ^]^ Reduced N limitation lowers CUE_ST_, increasing SOC through POC accumulation and MAOC conversion. The SEM confirmed this mechanism: microbial energy shifted from N acquisition (lower CUE_ST_) to biomass production and C mineralization, enhancing MAOC formation.^[^ ^26^ ^]^ Despite the high CUE_ST_ in degraded soils, low productivity limited stable SOC formation due to insufficient C inputs. Soil moisture limitations in degraded grasslands further elevated CUE_ST_ by suppressing metabolic rates.^[^ ^43^ ^]^

Higher plant productivity and root biomass under active restoration likely contributed additional organic substrates to microbes, linking vegetation recovery with microbial SOC stabilization.^[^ ^44^ ^]^ For example, the presence of leguminous species or improved N cycling in restored sites may have provided N inputs and C substrates, although we did not directly measure N fixation or plant traits in this study. These enhanced inputs and nutrient availability can help explain the observed rise in microbial biomass and stable SOC pools. Notably, POC displayed smaller gains, highlighting MAOC's critical role in stabilizing SOC.^[^ ^45^ ^]^ The increase in microbial biomass and the development of more stable SOC pools, particularly MAOC, were in line with the decrease in CUE_ST_. The accumulation of POC and its subsequent conversion into MAOC were due to the increasing microbial biomass,^[^ ^45^ ^]^ which also helped to increase SOC stocks overall. Active restoration alleviated N limitations, likely linked to increased N fixation rates. Although POC is generally more sensitive to N availability than MAOC,^[^ ^46^, ^47^ ^]^ active restoration predominantly increased SOC through MAOC.

This contrasts with N‐addition studies on the QTP, which reported greater increases in POC than MAOC.^[^ ^48^ ^]^ We propose that this discrepancy between studies arises from fundamental differences in ecosystem responses between direct N‐input and restoration practices.^[^ ^49^ ^]^ N‐addition stimulates mainly labile C inputs through enhanced plant productivity and root exudation, resulting in POC accumulation; while active restoration establishes self‐sustaining nutrient cycles by enhancing microbial biomass, reducing CUE_ST_, and accelerating the transformation of POC into MAOC, ultimately promoting long‐term stabilization of soil C sequestration.^[^ ^14^ ^]^ The transition from severe to mild N limitation under restoration shifts microbial contributions toward stable MAOC. This implies that the availability of nutrients, especially N, is essential for controlling the efficiency of microbial C consumption and, in turn, SOC dynamics.^[^ ^16^ ^]^ However, these processes may change with different ecosystem contexts as the balance between CUE, N limitation, and SOC stabilization can be influenced by soil texture, climate, and vegetation type.

### 3.3 Differential Responses of POC and MAOC to Restoration Strategies

POC and MAOC responded differently to restoration approaches, reflecting distinct roles in soil C cycles. Active restoration increased both fractions, but MAOC increased to a greater extent, which indicates that microbial stabilization processes responded strongly to restoration than POC. Vegetation recovery modifies rhizosphere microbiota, likely via enhanced symbiotic N fixation and enzymatic conversion of POC to MAOC.^[^ ^50^ ^]^

By contrast, passive restoration reduced microbial N limitation only slightly but failed to enhance SOC stocks as POC and MAOC did not increase.^[^ ^51^, ^52^ ^]^ The slow natural succession may have failed to boost plant productivity and microbial activity sufficiently in the nutrient‐poor soils. Random forest modelling supported these findings, identifying soil BD, CUE_ST_, and enzyme vector angle as the primary factors influencing SOC stock under active restoration. POC, MBC, and MAOC followed in importance; however, these factors did not lead to increases in SOC stocks under passive restoration. Under passive restoration, enzyme angle and POC had the greatest relative importance, but since overall SOC did not increase, changes in these factors were insufficient to build soil C stock. In particular, soil BD played an important role under active restoration:^[^ ^53^ ^]^ decreased BD can improve soil physical conditions and increase C inputs, but under passive restoration, BD remained relatively unchanged, limiting its influence on SOC.

### 3.4 Study Implications and Limitations

Our results have important implications for the restoration and management of degraded grasslands. In the short to medium term, active restoration strategies (such as planting, fertilization, or other interventions) may be more effective than passive recovery for sequestering soil C. By alleviating microbial nutrient limitations and boosting microbial processing of organic matter into stable C pools, active restoration can accelerate soil C accumulation. These findings can benefit the development of conservation and land management policies aiming to maximize C sequestration and ecosystem recovery.

We also acknowledge several limitations. First, the 10‐year duration of the restoration may be relatively short to capture the full effects of passive restoration, which often requires longer periods for changes in SOC stock. Thus, passive restoration might eventually lead to larger SOC gains than observed here. Long‐term restoration would be needed to determine whether SOC stocks in passive restoration sites eventually equal those of active sites. Second, we did not directly measure vegetation traits (e.g., plant productivity, species composition, litter quality) or biological N fixation rates, which are known to influence SOC inputs. Future work quantifying these plant‐driven factors would help disentangle their effects from microbial processes. Third, our method for assessing microbial nutrient limitation was based on ratios of potential enzyme activities, which provides only a qualitative index. Enzyme assays measure potential activity under laboratory conditions, not actual in situ rates, and the stoichiometric vector approach simplifies complex nutrient demands.^[^ ^54^ ^]^ These methodological uncertainties mean that any quantitative interpretation of N limitation relief should be made with caution. Finally, our findings are specific to the alpine grassland ecosystem. Soil texture, climate, and restoration practices can all influence microbial responses and C stabilization, so applying these results to other locations should be done carefully. Nevertheless, despite these caveats, our analyses of SOC fractions, enzyme activity, and microbial CUE provide a useful framework for understanding how different restoration strategies impact soil C.

## 4 Conclusion

Active restoration for over 10 years alleviated microbial nitrogen limitation substantially and increased SOC stocks in the degraded alpine grassland. The latter occurred mainly through large gains in MAOC, which is the more stable soil C pool. By reducing microbial N limitation, active restoration enhanced microbial processing of organic inputs and promoted the transfer of POC into MAOC. Active restoration also increased extracellular enzyme activities related to C, N, and phosphorus cycling and reduced CUE_ST,_ changes that are consistent with greater microbial processing of organic inputs and with enhanced transfer of POC into MAOC. Passive restoration resulted in little change in SOC over the 10 years, but passive recovery can require decades to centuries in some systems to increase SOC. Where is a management priority. Active restoration can be effective for rapid soil C gains, but managers should weigh benefits against costs and local conditions. We recommend continued, long‐term monitoring and further measurements of plant productivity, N sources such as biological fixation, and soil mineralogy to better resolve the long‐term effectiveness of active versus passive restoration for soil C sequestration.

## 5 Experimental Section

### Location, Vegetation, and Climatic Conditions of Study Area

The study was carried out in Zoigê County, Sichuan Province, China (33°42’N, 102°29’E; 3,410 m above sea level), which lies on the eastern margin of the Tibetan Plateau. This region was selected as a representative alpine grassland ecosystem with severe desertification due to overgrazing and climate change, making it a critical area for studying restoration impacts on soil C dynamics. The area was characterized by a plateau cold‐temperate humid monsoon climate, with an average yearly temperature of 1.1 °C and no frost‐free days during the year. January was the coldest month with a daily mean of −10.6 °C, and July was the hottest month with a daily mean of 10.8 °C. Annual rainfall averages 600 mm, with 86% falling during the wet season from May to October.^[^ ^55^ ^]^ Subalpine meadow soil was the dominant soil type, but also contains marshy meadow soil, alpine cold desert soil, windblown sandy soil and rock‐forming soil. The soil type was Arenosol, as classified by the World Reference Base,^[^ ^56^ ^]^ and has a loose structure, with low resistance to wind erosion. In desertified areas, the soil texture was predominantly sandy, with particle size consisting of ≈96% sand, 1–2% silt, and 2–4% clay.^[^ ^57^, ^58^ ^]^ Grassland vegetation was comprised mainly of Gramineae and Cyperaceae, which together cover ≈80% of the area. The most abundant species include *Kobresia pygmaea*, *K. humilis*, *Elymus nutans*, *Potentilla anserina*, *Carex scabrirostris*, and *Oxytropis ochrocephala*.

### Field Site

The study used a stratified random sampling design to establish nine independent 10 m × 10 m plots, with three replicate plots in each of the three grassland types: degraded, passively restored, and actively restored. Plots within the same grassland type were separated by a minimum distance of ≥200 m to ensure spatial independence and minimize potential autocorrelation effects. Desertified grasslands at each site underwent active and passive regeneration, while part of the degraded grassland was preserved to enable comparison with the restored grasslands. To ensure that sampling locations were not influenced by factors such as site‐specific conditions, climate, or elevation, the study sites were selected to maintain consistency in elevation and climatic conditions. Prior to restoration, all restored sites underwent severe desertification, and grasslands currently experiencing these conditions were selected as degraded sites. Passive restoration enables degraded grasslands to recover with minimal human intervention by using sand barriers to block sand flow, a process that was initiated in 2012. In active restoration, the grasslands were seeded with dominant native grasses from late April to early May 2012 at a rate of 0.1 kg ha^−1^ for *Elymus sibiricus*, 0.1 kg ha^−1^ for *Elymus nutans*, 0.1 kg ha^−1^ for *Melilotoides ruthenicus*, and 0.3 kg ha^−1^ for *Avena sativa*. Generally, no further seeding was required in the first year. Fencing excluded livestock during the seeding year, with winter grazing permitted in subsequent years following an assessment of grass establishment. Degraded sites were subjected to unrestricted grazing, while passively restored and actively restored grasslands were used as winter pastures at stocking densities of 3.3–5.2 sheep units per hectare. For detailed information on the three types of grasslands, see Table S1 (Supporting Information).

### Soil Sampling

In August 2022 (the peak growing season), five soil cores were collected, each at 0–15 cm and 15–30 cm from random points within each plot, and were subsequently combined into one sample per depth per plot. This compositing integrates within‐plot spatial variability while maintaining plot‐level biological replicates (*n* = 3 per grassland type) for statistical analysis.^[^ ^59^, ^60^, ^61^ ^]^ Samples were sieved through a 2‐mm mesh to remove litter, stones, and debris, and subsequently were separated into two subsamples. The first subsample was air‐dried for physicochemical analyses, while the second was stored at 4 °C to determine microbial biomass carbon (MBC) and microbial biomass nitrogen (MBN).

### Soil C Stocks and Physicochemical Analysis

Soil was oven‐dried at 105°C for 48 h to determine moisture content; BD was calculated by dividing the soil's dry weight by its bulk volume; Soil pH was quantified by a glass electrode in a 1:2.5 soil–deionized water (w/v) mixture;^[^ ^62^ ^]^ and SOC was measured by the potassium dichromate oxidation procedure of Walkley and Black.^[^ ^63^ ^]^ POC and MAOC concentrations were determined following Cambardella et al. (2022).^[^ ^64^ ^]^ Twenty g of air‐dried soil were centrifuged in a 5% sodium hexametaphosphate solution at 180 rpm for 2 h and were then sieved through a 53 µm mesh. The material that remained (>53 µm) was defined as POC, while that passing through (<53 µm) was defined as MAOC. Soil C stocks were calculated following Deng et al. (2014)^[^ ^65^ ^]^ as:

(1) $$\begin{array}{c} \mathrm{Soil}\;C\;\mathrm{stock}\;\left(\mathrm{kg}\;m^{-2}\right)=\mathrm{SOC}\;\left(\%\right)\times \mathrm{BD}\;\left(\mathrm{kg}\;m^{-3}\right)\times \mathrm{soil}\;\mathrm{depth}\;\left(m\right) \end{array}$$

The chloroform fumigation‐extraction technique was used to measure the soil MBC and MBN levels.^[^ ^66^ ^]^ Twenty g of fresh soil were fumigated in a vacuum dryer for 24 h in the dark with ethanol‐free chloroform. Fumigated and non‐fumigated samples were then treated and shaken with 0.5 m K_2_SO_4_ for 1 h, after which time they were filtered with a 0.45‐µm membrane. TDN and dissolved organic C (DOC) were quantified as the amounts of N and organic C extracted from non‐fumigated soil, respectively.^[^ ^66^ ^]^ MBC and MBN were calculated as the difference between the organic C and N levels in soil that underwent fumigation and soil that did not.^[^ ^67^ ^]^

The activities of six extracellular enzymes, including cellobiohydrolase (CBH) and β‐glucosidase (βG) for C acquisition,^[^ ^68^ ^]^ β‐N‐acetylglucosaminidase (NAG), leucine aminopeptidase (LAP), and urease (UE) for N acquisition, and acid/alkaline phosphatase (AP) for P acquisition,^[^ ^69^ ^]^ were measured. βG, CBH, and NAG activities were determined using *p*‐nitrophenyl‐β‐D‐glucopyranoside, 4‐nitrophenyl‐β‐D‐cellobioside, and N‐acetyl‐D‐glucosamine as substrates, respectively. Following an hour's incubation at 37 °C, the p‐nitroaniline concentration was measured colorimetrically at 405 nm, and LAP activity was determined using L‐leucine‐p‐nitroanilide as the substrate. After a 24 h incubation at 30 °C, the p‐nitroaniline concentration was measured colormetrically at 405 nm. Soil AP activity was determined using disodium phenyl phosphate as a substrate. After incubation for 24 h at 37 °C, the phenol concentration was determined at 660 nm. Urea was used as the substrate to determine UE activity. Following incubation for 24 h at 37 °C, ammonia concentration was measured colorimetrically at 578 nm. Enzyme activities were determined with commercial assay kits (Solarbio Science and Technology, Beijing, China) following the manufacturer's instructions.

To date, CUE has been measured mainly by using labelled substrate approaches in culture‐based experiments. However, resource manipulation or addition in culture studies may alter the native microbial community structure and function, leading to cascading effects on microbial metabolism that can bias CUE measurements.^[^ ^26^, ^70^ ^]^ Therefore, to estimate the intrinsic differences in CUE across grassland types, the CUE_ST_ model was used based on a stoichiometric model. Microbial CUE_ST_ was determined with C:N stoichiometry as follows:

(2) $$\mathrm{CU}E_{\mathrm{ST}}=\mathrm{CU}E_{\max}\left[S_{C:N}/\left(S_{C:N}+K_{N}\right)\right]$$

(3) $$S_{C:N}=\left(1/\mathrm{EE}A_{C:N}\right)\times \left(B_{C:N}/R_{C:N}\right)$$

where *CUE_ST_* was specified as the microbial C use efficiency, and *S_C:N_* was treated as a scalar quantifying the extent to which enzyme activities redressed disparities between resource ratios and biomass. The half‐saturation constant *K_N_*, was assigned a value of 0.5. *CUE_max_* served as the upper threshold for growth efficiency and was defined as 0.6 based on thermodynamic constraints.^[^ ^25^ ^]^ *EEA_C:N_* was derived as the ratio of C‐related to N‐related enzyme activities, calculated by βG/(NAG + LAP). *R_C:N_* was calculated as dissolved C divided by dissolved N.^[^ ^71^ ^]^

Microbial nutrient limitation was assessed based on ecological enzyme activity stoichiometry. It was important to note that vector analysis provides only a relative measure of nutrient limitation: longer vector lengths indicate greater C limitation, vector angles greater than 45° indicate P limitation, while angles less than 45° indicate N limitation.^[^ ^27^ ^]^ These metrics were calculated using the following equations:

(4) $$\begin{array}{ccc} \mathrm{Vector}\;\mathrm{angle} & = & \mathrm{Degrees}\;\left\{\mathrm{ATAN}2\left[\left(\ln\;\left(\beta G\;+\;\mathrm{CBH}\right)\;/\ln\mathrm{AP}\right),\ln\;\left(\beta G\;+\;\mathrm{CBH}\right)\;/\right.\right.\\ & & \left.\left.\ln\left(\mathrm{NAG}+\mathrm{LAP}+\mathrm{UE}\right)\right]\right\} \end{array}$$

(5) $$\mathrm{Vector}\;\mathrm{length}=\sqrt{\begin{array}{c} {\left[\ln\left(\beta G+\mathrm{CBH}\right)/\ln\left(\mathrm{NAG}+\mathrm{LAP}+\mathrm{UE}\right)\right]}^{2}\\ \;+\;{\left[\;\ln\beta G/\mathrm{lnAP}\;\right]}^{2} \end{array}}$$

### Statistical Analyses

All values were presented as means + SE. Residual normality for each response variable was tested by the Shapiro–Wilks test to validate parametric conditions. A square‐root transformation was applied to skewed data. A one‐way ANOVA compared SOC stocks and microbial parameters among grassland types, with Tukey's method separating means. Pearson's correlation tested relationships between soil microbial CUE_ST_, nutrient limitation (vector angle and length), and SOC stocks using R statistical software (v. 4.3.0). To identify key predictors of SOC stocks and rank their importance, a random forest analysis was implemented with 1000 decision trees. Significance of predictors was assessed at the *p* < 0.05 level. Structural equation modelling (SEM) tested hypothesized causal pathways linking soil microbial N limitation (enzyme vector angle and vector length), microbial CUE, MAOC, POC, and SOC stock. The maximum likelihood method compared the variance‐covariance matrix implied by the SEM with the observed matrix. Model parameters were estimated using AMOS 24 (AMOS Development Corporation, Chicago, IL, USA). Goodness‐of‐fit was evaluated via chi‐square statistics, where non‐significant *p‐*values (*p* > 0.05) indicated consistency between model predictions and empirical data.^[^ ^72^ ^]^

## Conflict of Interest

The authors declare no conflict of interest.

## Author Contributions

J.G., F.S., and J.W. contributed equally to this work. J.G. contributed to writing the original draft, writing – review and editing, visualization, methodology, investigation, formal analysis, and data curation. F.S. contributed to writing – review and editing, and conceptualization. J.W. contributed to writing – review and editing, investigation, and data curation. S.Z., Z.M., J.Z., L.L., J.C., and A.E. contributed to writing – review, and editing. Y.X., L.L., and T.K. contributed to the investigation. Y.C., J.S., and J.P. contributed to writing – review and editing, and methodology, and Y.B. contributed to writing – review and editing, conceptualization, funding acquisition, and project administration.

## Supporting information



Supporting Information

## Data Availability

The data that support the findings of this study are available from the corresponding author upon reasonable request.
