# Supplementary material for: Long‐Term Active Rather than Passive Restoration Promotes Soil Organic Carbon Accumulation by Alleviating Microbial Nitrogen Limitation in an Extremely Degraded Alpine Grassland
Source: Adv Sci (Weinh). 2025 Nov 29;13(9):e10549. doi: 10.1002/advs.202510549 (PMC12903966; doi:10.1002/advs.202510549)
Supplement: Supplementary file 1 — Supporting Information [file ADVS-13-e10549-s001.docx]

Supporting Information

Long-term active rather than passive restoration promotes soil organic carbon accumulation by alleviating microbial nitrogen limitation in an extremely degraded alpine grassland

Jinchao Gong, Feida Sun, Jingjing Wu, Shijie Zhou, Yue Xiu, Linlin Li, Tahmina Kausar, Zhouwen Ma, Jiqiong Zhou, Lin Liu, Yongxing Cui, Jordi Sardans, Josep Peñuelas, Ahmed Elrys, Ji Chen, Yanfu Bai^*^


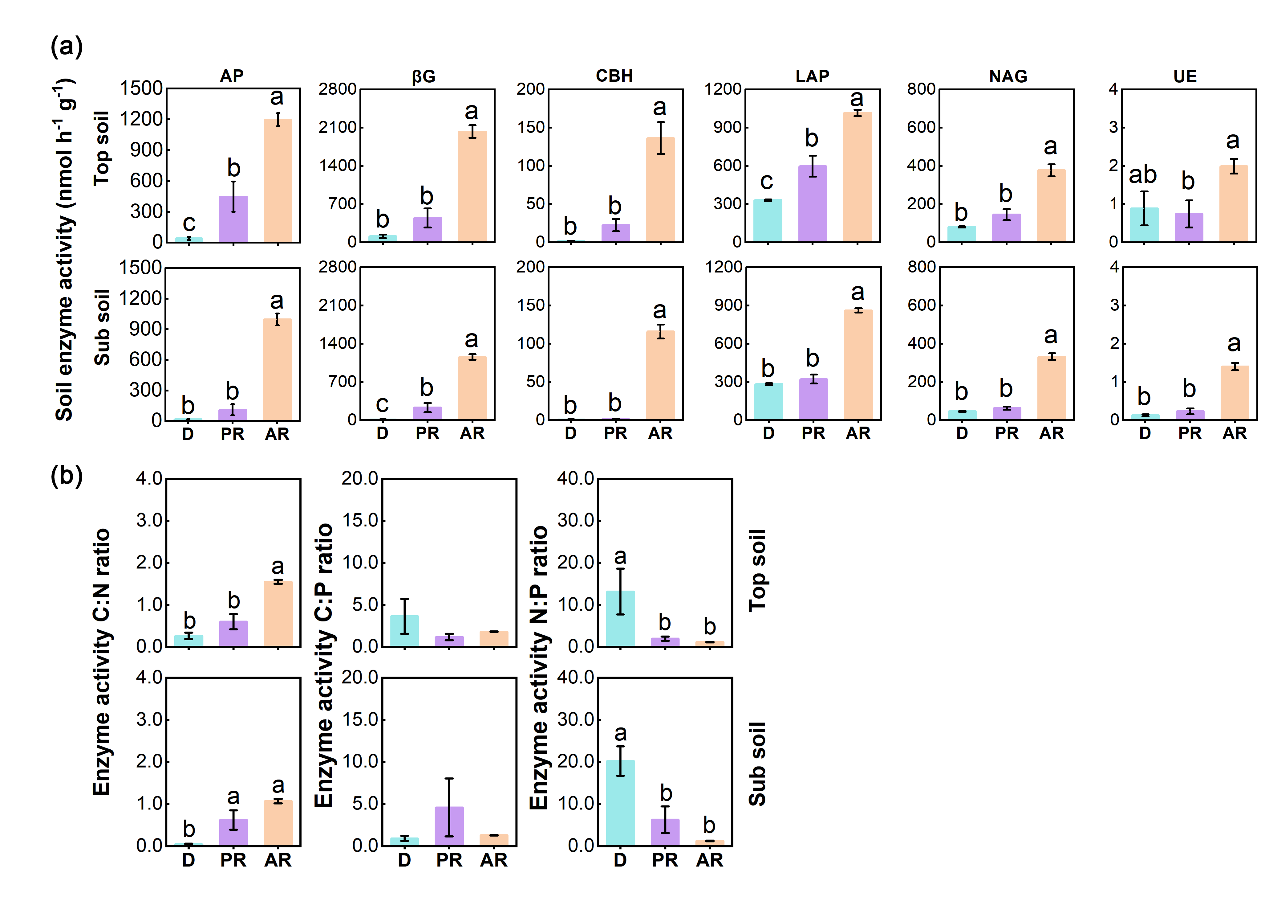


**Figure S1:** The effects of active and passive grassland restoration on (a) soil enzyme activities and (b) soil enzyme stoichiometric ratios (mean ± SE). Different letters indicate significant differences between means in different grassland types at *p* < 0.05. D: degraded, PR: passive restoration, AR: active restoration.

**Table S1** Study site information

| Study site | Position  (N, E) | Elevation (m) | Dominant vegetation species | Cover (%) | Management history |
| --- | --- | --- | --- | --- | --- |
| Degradation | 102°29'29''E 33°42'59''N | 3415 | *Equisetum arvense*; *Carex sabulosa* | 5-10 | Free grazing has been ongoing |
| Passive restoration | 102°29'41''E 33°42'44''N | 3417 | *Anaphalis lacteal*; *Carex tristachya*; *Stipa purpurea*; *Vicia sepium* | 70-84 | It was a desert until 2012. Grazing is prohibited for one year following restoration to allow vegetation to restore, after which grazing takes place in winter |
| Active restoration | 102°29'01''E 33°48'52''N | 3408 | *Artemisia frigida*; *Anaphalis lacteal*; *Artemisia annua*; *Melissitus ruthenica*; *Elymus nutans* | 95-99 | It was a desert until 2012. Grazing is prohibited for one year following restoration to allow vegetation to restore, after which grazing takes place in winter |

**Table S2** Comparison of soil physicochemical properties in topsoil (0–15 cm) and subsoil (15–30 cm) under degradation, passive restoration, and active restoration

| Soil properties | Soil Depth | Degradation | Passive Restoration | Active Restoration |
| --- | --- | --- | --- | --- |
| Soil bulk density  (g cm^-3^) | Topsoil | 1.47±0.04 a | 1.23±0.06 ab | 1.19±0.11 b |
|  | Subsoil | 1.48±0.02 a | 1.48±0.07 a | 1.12±0.07 b |
| Soil Moisture  (%) | Topsoil | 4.56±0.43 b | 5.37±0.41 b | 17.69±4.53 a |
|  | Subsoil | 4.01±0.25 | 3.56±0.46 | 14.19±6.66 |
| Soil pH | Topsoil | 7.58±0.06 c | 7.79±0.02 b | 7.95±0.05 a |
|  | Subsoil | 7.57±0.03 b | 7.87±0.02 a | 7.92±0.10 a |
| MBC  (mg kg^-1^) | Topsoil | 103.2±28.99 b | 123.6±48.25 b | 600.5±28.12 a |
|  | Subsoil | 79.9±29.08 b | 107.7±30.85 b | 519.6±61.78 a |
| MBN  (mg kg^-1^) | Topsoil | 1.19±0.65 c | 8.61±2.21 b | 76.40±2.04 a |
|  | Subsoil | 4.54±1.14 b | 4.79±0.56 b | 45.05±6.56 a |
| DOC  (mg kg^-1^) | Topsoil | 34.0±7.62 | 84.9±30.76 | 72.1±2.92 |
|  | Subsoil | 33.3±8.90 b | 25.4±1.73 b | 68.7±3.13 a |
| TDN  (mg kg^-1^) | Topsoil | 3.77±0.10 | 6.30±1.23 | 5.99±0.69 |
|  | Subsoil | 2.15±0.30 b | 2.60±0.19 b | 5.81±0.16 a |

**Note:** MBN: soil microbial biomass N, MBC: soil microbial biomass C, TDN: soil total N, DOC: soil dissolved organic C. Significant variations between means under various restoration approaches at *p* < 0.05 are indicated by different letters.
